# Efficacy and safety of postoperative adjuvant HAIC combining lenvatinib with or without PD-1 inhibitors in solitary large HCC: A multicenter retrospective study

**DOI:** 10.3389/fimmu.2025.1609352

**Published:** 2025-08-27

**Authors:** Yuxin Liang, Ming Wang, Deyuan Zhong, Hongtao Yan, Yuhao Su, Xing Chen, Xiaolun Huang, Zhengwei Leng

**Affiliations:** ^1^ Department of Liver Transplantation Center and HBP Surgery, Sichuan Clinical Research Center for Cancer, Sichuan Cancer Hospital and Institute, Sichuan Cancer Center, School of Medicine, University of Electronic Science and Technology of China, Chengdu, China; ^2^ Department of Hepatobiliary-Pancreatic Surgery, Cell Transplantation Center, Sichuan Provincial People’s Hospital, University of Electronic Science and Technology of China, Chengdu, China; ^3^ State Key Laboratory of Quality Research in Chinese Medicine, Macau Institute for Applied Research in Medicine and Health, Macau University of Science and Technology, Taipa, Macao, Macao SAR, China; ^4^ Department of Hepatobiliary Surgery II, Affiliated Hospital of North Sichuan Medical College, Nanchong, China

**Keywords:** solitary large hepatocellular carcinoma, postoperative adjuvant therapy, hepatic arterial infusion chemotherapy, PD-1 inhibitors, combined therapy

## Abstract

**Purpose:**

To evaluate the efficacy and safety of postoperative adjuvant hepatic arterial infusion chemotherapy (PA-HAIC) combined with lenvatinib and PD-1 inhibitors versus PA-HAIC with lenvatinib alone in patients with solitary large hepatocellular carcinoma (HCC, >5 cm).

**Methods:**

A total of 183 patients who underwent curative resection and subsequent PA-HAIC plus lenvatinib (HAIC-L, n = 108) or PA-HAIC combined with lenvatinib and PD-1 inhibitors (HAIC-L-P, n = 75) were enrolled from three centers between April 2021 and April 2023. Propensity score matching (PSM) was applied to balance baseline characteristics. Disease-free survival (DFS) and overall survival (OS) were analyzed using the Kaplan–Meier method and Cox proportional hazards models, while treatment-related adverse events (TRAEs) were compared between groups.

**Results:**

The HAIC-L-P group demonstrated significantly improved DFS compared to the HAIC-L group both before (HR: 0.570; P = 0.007) and after PSM (HR: 0.518; P = 0.018). In contrast, no statistically significant difference was observed in OS between the two groups. Multivariate analysis identified elevated AFP (≥400 ng/mL), microvascular invasion, and treatment strategy (HAIC-L *vs*. and HAIC-L-P) as independent predictors of DFS. Additionally, the overall safety profiles were comparable, with similar incidences of TRAEs and no significant increase in hepatic toxicity with PD-1 inhibitor addition.

**Conclusion:**

PA-HAIC combined with lenvatinib and PD-1 inhibitors significantly enhances DFS in patients with solitary large HCC, offering a promising adjuvant approach with acceptable safety. Further prospective, biomarker-driven trials are warranted to validate these findings and optimize patient selection.

## 1 Introduction

Hepatocellular carcinoma (HCC) remains a significant global health burden, ranking as the fourth leading cause of cancer-related mortality worldwide (1). While surgical resection, ablation, and liver transplantation could offer curative potential for early-stage HCC (2, 3), solitary large HCC (tumor diameter >5 cm) remains a therapeutic challenge due to its aggressive biology and high postoperative recurrence rates, even after curative resection (4–7). Tumor size often serves as an independent prognostic factor in HCC, with larger tumors correlating with increased vascular invasion, rapid progression, and diminished survival (8, 9).

Nowadays, postoperative adjuvant hepatic arterial infusion chemotherapy (PA-HAIC) has emerged as a viable option for HCC patients with high-risk features, including microvascular invasion (MVI), and huge single HCC (10–12). By delivering high-dose chemotherapeutic agents directly to the liver, PA-HAIC targets residual micrometastases and circulating tumor cells, potentially delaying recurrence (13). Emerging evidence have also indicated that combining HAIC with tyrosine kinase inhibitors (TKIs) such as lenvatinib (a multi-targeted antiangiogenic agent) may enhance therapeutic efficacy by suppressing angiogenesis and tumor regrowth (14, 15). Furthermore, immune checkpoint inhibitors, particularly programmed death-1 (PD-1) inhibitors, have shown synergistic antitumor effects when combined with TKIs and HAIC in advanced HCC, presumably by modulating the immunosuppressive tumor microenvironment and extending patient survival (16, 17).

Despite these promising advances, the triple-modality regimen of PA-HAIC, lenvatinib, and PD-1 inhibitors remains unexplored in the adjuvant management of solitary large HCC. Moreover, overlapping toxicities from chemotherapy (HAIC), antiangiogenic agents (lenvatinib), and immunotherapy (PD-1 inhibitors) necessitate rigorous safety evaluations, particularly in postoperative patients with compromised liver function. Addressing these gaps is imperative, as solitary large HCC represents a high-risk subgroup with limited therapeutic options and disproportionately poor outcomes.

This multicenter retrospective study aimed to evaluate the efficacy and safety of PA-HAIC combined with lenvatinib and PD-1 inhibitors versus PA-HAIC plus lenvatinib alone in patients with solitary large HCC after curative resection. By comparing disease-free survival (DFS), overall survival (OS), and adverse events between the two groups, this study seeks to provide evidence for optimizing adjuvant strategies in this high-risk population.

## 2 Materials and methods

### 2.1 Patient cohort and study design

**Figure 1:**
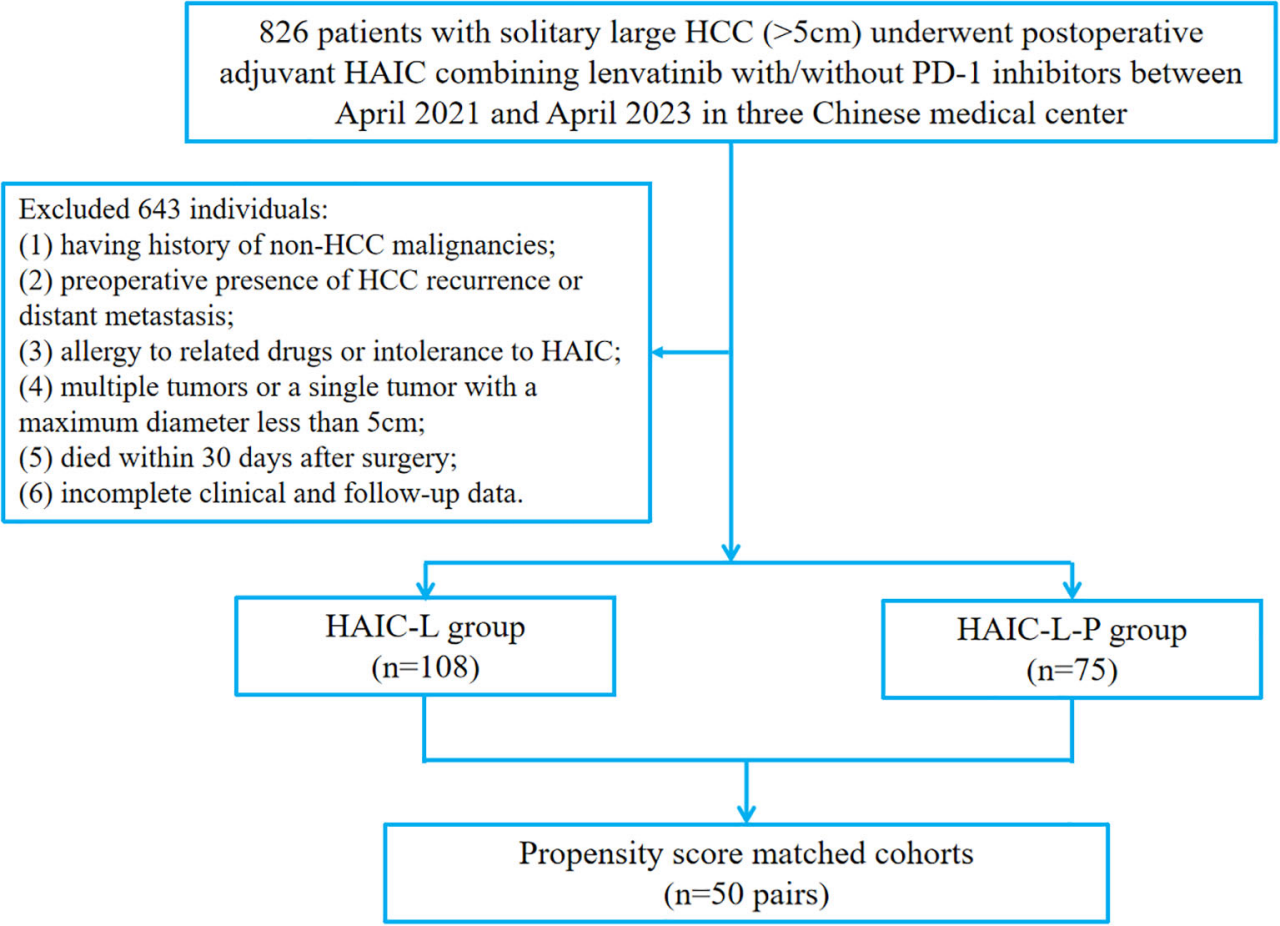
The flowchart of patient enrollment. HCC, hepatocellular carcinoma; HAIC, hepatic arterial infusion chemotherapy; PD-1, programmed cell death protein 1.

This retrospective study enrolled patients with solitary large HCC (>5cm) who underwent PA-HAIC combined with lenvatinib or PA-HAIC combined with lenvatinib and PD-1 inhibitors at Sichuan Cancer Hospital, Sichuan Provincial People’s Hospital, and the Affiliated Hospital of North Sichuan Medical College from April 2021 to April 2023. The inclusion criteria were as follows: (1) histologically confirmation of HCC; (2) Eastern Cooperative Oncology Group performance status of 0 or 1; (3) no prior or concomitant anticancer therapy; (3) R0 surgical resection with curative intent; (4) solitary tumor >5 cm in diameter; (5) adjuvant PA-HAIC with lenvatinib ± PD-1 inhibitor as the only postoperative therapy. The exclusion criteria included: (1) having history of non-HCC malignancies; (2) preoperative evidence of HCC recurrence or distant metastasis; (3) multiple tumors or a solitary tumor ≤5 cm; (4) drug allergy or intolerance to HAIC; (5) postoperative death within 30 days; (6) incomplete clinicopathological or follow-up data. A total of 183 eligible patients were included into the study. The study design is illustrated in **Figure 1**.

The study protocol was approved by the Human Ethics Committee of Sichuan Cancer Hospital. All procedures complied with the principles of the Helsinki Declaration. At the time of treatment, all patients provided written informed consent for their clinical data to be used in scientific researches (including retrospective studies).

### 2.2 Follow up

Patients were followed up every 1–2 months during the first postoperative year and every 3 months thereafter if no recurrence or metastasis was detected. Follow-up evaluations included laboratory tests and imaging via computed tomography (CT) or magnetic resonance imaging (MRI). The primary endpoint was disease-free survival (DFS), defined as the time from surgery to recurrence, metastasis, or death from any cause. The secondary endpoint was overall survival (OS), defined as the time from surgery to death from any cause. Patients without recurrence, metastasis, or death by the end of follow-up (April 2024) were censored as alive and event-free.

### 2.3 Clinicopathological data collection

Clinicopathological data potentially related to prognosis were collected within 7 days prior to surgery, including demographic characteristics (age, sex, body mass index [BMI]), laboratory parameters (serum biomarkers, liver function tests, coagulation profile, and hepatitis B virus markers), and tumor-related features. Tumor characteristics included histopathological type, presence of cirrhosis, tumor diameter, number of nodules, and MVI. MVI was defined as the presence of cancer cell clusters within a vascular lumen lined by endothelial cells, observable only under microscopy (18).

### 2.4 Treatment

All patients initiated adjuvant therapy 4–6 weeks after surgery. Treatment consisted of either PA-HAIC with lenvatinib or in combination with PD-1 inhibitors. HAIC was performed based on established protocols (10, 19). Each patient received 1–3 cycles of HAIC with 4-week intervals. Treatment efficacy and toxicity were monitored regularly via imaging and clinical assessments. Therapy was discontinued in the event of unacceptable adverse effects, patient withdrawal, or disease progression. PD-1 inhibitors (Sintilimab) were administered intravenously at a fixed dose of 200 mg every three weeks, with dose modifications as per toxicity management guidelines provided by the manufacturer.

### 2.5 Statistical analysis

Continuous variables were expressed as medians with interquartile ranges (IQRs) or mean ± standard deviation (SD), while categorical variables were reported as counts and percentages. Categorical variables were compared using the Chi-square or Fisher’s exact test, and continuous variables were compared using the Student’s *t* test or Mann–Whitney *U* test, as appropriate. To adjust for baseline confounding between groups, propensity score matching (PSM) was conducted using a 1:1 nearest-neighbor algorithm with a caliper width of 0.05. Variables included in the propensity score model were age, gender, and albumin (ALB). DFS and OS were analyzed using the Kaplan–Meier method, and survival curves were compared using the log-rank test. Univariate and multivariate Cox proportional hazards models were employed to identify independent prognostic factors. Variables with P < 0.05 in univariate analysis were included in the multivariate model. All statistical analyses were performed using SPSS software version 22.0 (IBM Corp., Armonk, NY, USA). A two-sided P value < 0.05 was considered statistically significant.

## 3 Results

### 3.1 Patient characteristics

A total of 183 patients with solitary large HCC (>5cm) were included, including 108 patients in the HAIC-L group and 75 in the HAIC-L-P group. The clinicopathological characteristics before and after PSM are summarized in **Table 1**. Of the entire cohort, 156 patients (85.2%) were male, with a median age of 52 years. Hepatitis B virus (HBV) infection was present in 136 patients (74.3%), and 144 patients (78.7%) were classified as Barcelona Clinic Liver Cancer (BCLC) stage A. Before PSM, significant differences were observed between the two groups in age (P = 0.022), gender (P = 0.036), and ALB levels (P = 0.041). After PSM, no significant differences remained, indicating that baseline characteristics were well-balanced between the two groups.

**Table 1:** Baseline characteristics of the HCC patients before and after PSM.

Characteristics	Before PSM			After PSM		
	HAIC-L (n=108)	HAIC-L-P (n=75)	P value	HAIC-L (n=50)	HAIC-L-P (n=50)	P value
Age, years	57 (47-66)	59 (54-67)	**0.022**	59 ± 10	59 ± 9	0.943
Gender			**0.036**			1.000
Male	97 (53%)	59 (32.2%)		46 (46%)	46 (46%)	
Female	11 (6%)	16 (8.7%)		4 (4%)	4 (4%)	
BMI, kg/m^2^	22.09 (20.58-23.66)	22.31 (20.26-24.64)	0.334	22.49 (20.83-23.97)	22.34 (20.10, 24.88)	0.850
Etiology of HCC			0.436			0.349
HBV	78 (42.6%)	58 (31.7%)		36 (36%)	40 (40%)	
Others	30 (16.4%)	17 (9.3%)		14 (14%)	10 (10%)	
BCLC stage			0.467			0.461
A	83 (45.4%)	61 (33.3%)		38 (38%)	41 (41%)	
C	25 (13.7%)	14 (7.7%)		12 (12%)	9 (9%)	
Child-Pugh class			0.191			0.640
A	74 (40.4%)	58 (31.7%)		37 (37%)	39 (39%)	
B	34 (18.6%)	17 (9.3%)		13 (13%)	11 (11%)	
ALT, U/L	29 (18-49)	33 (19-46)	0.663	33 (19-53)	34 (18-46)	1.000
ALB, g/L	37.4 (33.7-40.3)	39.1 (35.4-41.0)	**0.041**	38.7 (35.2-40.9)	39.1 (35.4-41.4)	0.639
Bilirubin, µmol/L	15.8 (11.5-22.23)	15.0 (9.9, 21.5)	0.391	17.9 (10.9-23.1)	14.1 (9.9-22.4)	0.221
Leukocyte count, 10^9^/L	5.80 (4.46-6.79)	5.18 (4.17-6.77)	0.226	5.57 (4.44-6.46)	5.36 (4.72-6.58)	0.524
Neutrophil count, 10^9^/L	3.62 (2.64-4.56)	3.45 (2.35-4.25)	0.160	3.35 (2.55-4.03)	3.52 (2.78-4.20)	0.542
Lymphocyte count, 10^9^/L	1.27 (1.00-1.60)	1.2 (0.94-1.70)	0.889	1.33 ± 0.53	1.40 ± 0.48	0.510
Platelet count, 10^9^/L	166 (114-218)	141 (95-199)	0.083	159 (107-203)	155 (98-199)	0.689
Prothrombin time, s	11.9 (11.1-12.7)	11.9 (11.4-12.9)	0.274	11.9 (11.2-12.6)	12.1 (11.4-12.9)	0.368
AFP, ng/mL	145.32 (6.54-1000)	129.02 (8.55-964.14)	0.897	103.11 (7.50-1000)	169.20 (8.82-982.07)	0.923
Tumor size (cm)	8.3 (6.5-11.1)	8.0 (6.5-11.0)	0.626	8.5 (6.6-10.4)	8.0 (6.5-10.5)	0.513
Histopathological type			0.331			0.656
Poorly differentiated	33 (18%)	18 (9.8%)		15 (15%)	13 (13%)	
Medium‐high differentiated	75 (41%)	57 (31.1%)		35 (35%)	37 (37%)	
MVI			0.210			0.317
No	46 (25.1%)	39 (21.3%)		22 (22%)	27 (27%)	
Yes	62 (33.9%)	36 (19.7%)		28 (28%)	23 (23%)	
Cirrhosis			0.669			0.221
No	37 (23%)	28 (15.3%)		23 (23%)	17 (17%)	
Yes	71 (36.1%)	47 (25.7%)		27 (27%)	33 (33%)	

HCC, hepatocellular carcinoma; PSM, propensity score matching; HAIC, hepatic arterial infusion chemotherapy; BMI, body mass index; HBV, hepatitis B virus; BCLC, Barcelona Clinic Liver Cancer; ALT, alanine transaminase; ALB, albumin; AFP, alpha-fetoprotein; MVI, microvascular invasion.

Bold values indicate statistically significant P values.

### 3.2 Survival analysis

The median follow-up duration was 21 months (interquartile range, 14–31 months). At the end of the follow-up, tumor progression was observed in 69 patients (63.9%) and 32 patients (29.6%) had died in the HAIC-L group. Moreover, 31 patients (41.3%) in the HAIC-L-P group experienced tumor progression, and 16 patients (21.3%) died.

**Figure 2:**
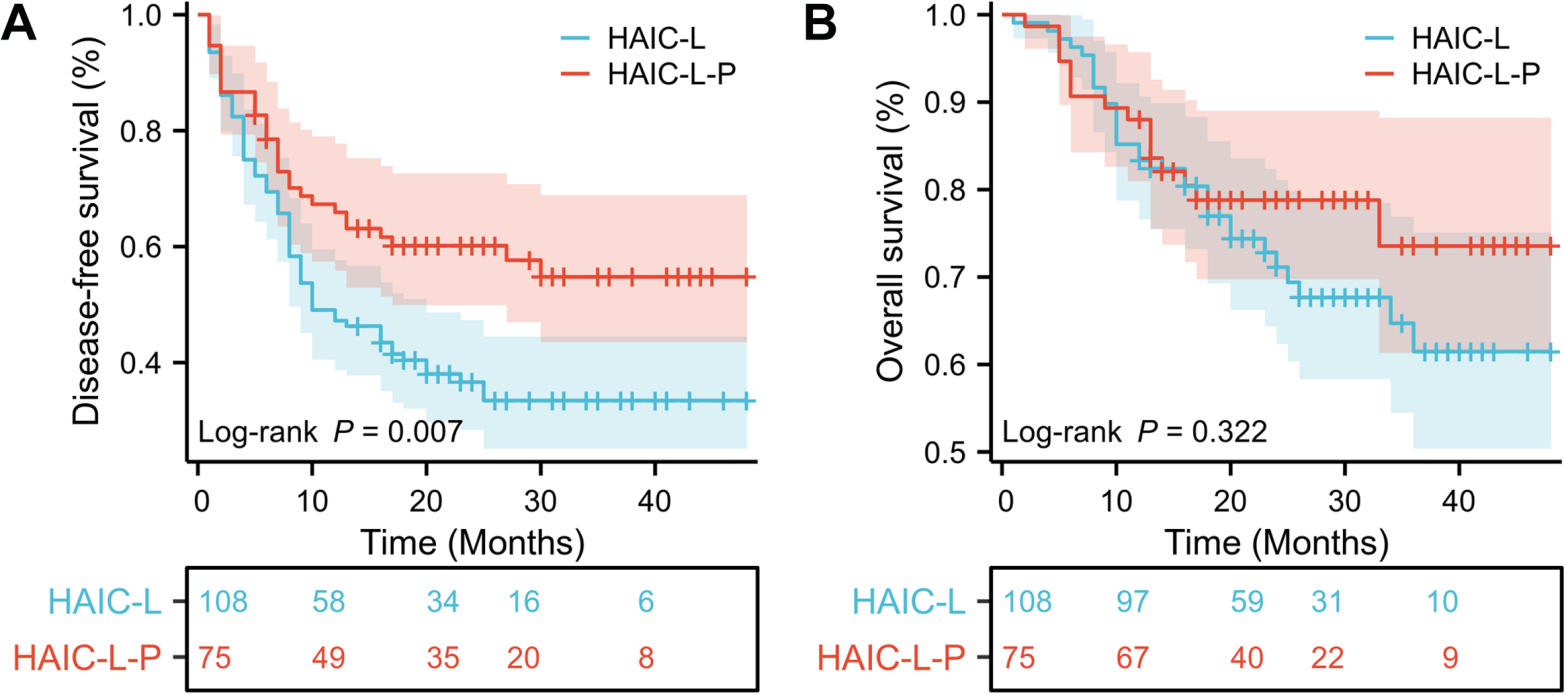
Kaplan-Meier survival curves of disease-free survival **(A)** and overall survival **(B)** for the patients with solitary large HCC in the two groups before PSM. HCC, hepatocellular carcinoma; PSM, propensity score matching; HAIC, hepatic arterial infusion chemotherapy.

**Figure 3:**
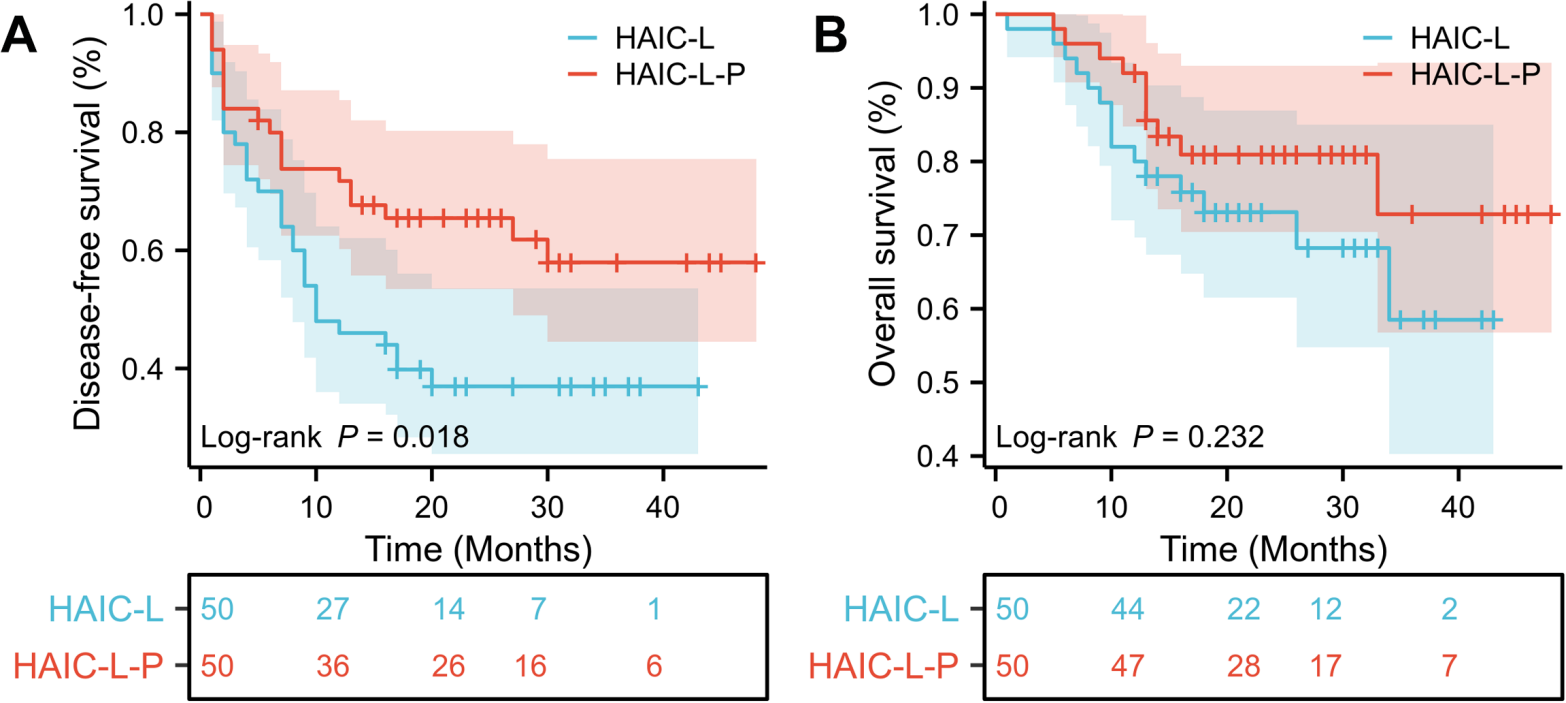
Kaplan-Meier survival curves of disease-free survival **(A)** and overall survival **(B)** for the patients with solitary large HCC in the two groups after PSM. HCC, hepatocellular carcinoma; PSM, propensity score matching; HAIC, hepatic arterial infusion chemotherapy.

Before PSM, DFS was significantly better in the HAIC-L-P group than in the HAIC-L group (hazard ratio [HR]: 0.570; 95% confidence interval [CI]: 0.384–0.846; P = 0.007; **Figure 2A**). After PSM, the HAIC-L-P group also demonstrate superior DFS (HR: 0.518; 95% CI: 0.297–0.903; P = 0.018; **Figure 3A**). However, no significant difference in OS was observed between the groups, both before and after PSM (P = 0.322; **Figure 2B**; P = 0.232; **Figure 3B**).

### 3.3 Analysis of independent prognostic factors

In the matched cohort, all variables were categorized and analyzed using univariate and multivariate Cox regression (**Table 2**). Univariate analysis revealed that alpha-fetoprotein (AFP, <400 ng/mL *vs*. ≥400 ng/mL, P = 0.001), microvascular invasion (MVI, no *vs*. yes, P = 0.013), and treatment strategy (HAIC-L *vs*. HAIC-L-P, P = 0.021) were significantly associated with DFS. For OS, significant predictors included alanine aminotransferase (ALT, <35 U/L *vs*. ≥35 U/L, P = 0.004) and AFP (<400 ng/mL *vs*. ≥400 ng/mL, P = 0.017). Multivariate analysis identified AFP (HR: 2.466; 95% CI: 1.405–4.326; P = 0.002), MVI (HR: 1.825; 95% CI: 1.022–3.259; P = 0.042), and treatment strategy (HR: 0.530; 95% CI: 0.297–0.946; P = 0.032) as independent prognostic factors for DFS. AFP (HR: 2.759; 95% CI: 1.216–6.261; P = 0.015) and ALT (HR: 3.873; 95% CI: 1.538–9.755; P = 0.004) were identified as independent predictors of OS.

**Table 2:** Univariate and multivariate Cox regression analyses of the predictors for disease-free survival and overall survival of the HCC patients after PSM.

Characteristics	DFS				OS			
	Univariate analysis		Multivariate analysis		Univariate analysis		Multivariate analysis	
	HR (95% CI)	P value	HR (95% CI)	P value	HR (95% CI)	P value	HR (95% CI)	P value
Age, years (<60 *vs*. ≥60)	0.569 (0.310 - 1.043)	0.068			0.866 (0.382 - 1.964)	0.731		
Bilirubin, µmol/L (<20 *vs*. ≥20)	0.690 (0.372 - 1.279)	0.239			0.836 (0.360 - 1.940)	0.677		
ALT, U/L (<35 *vs*. ≥35)	1.012 (0.581 - 1.762)	0.966			3.801 (1.515 - 9.536)	**0.004**	3.873 (1.538 - 9.755)	**0.004**
Histopathological type (Poorly *vs*. Medium-high)	0.602 (0.335 - 1.083)	0.090			0.555 (0.244 - 1.262)	0.160		
Cirrhosis (No *vs*. Yes)	1.062 (0.600 - 1.881)	0.837			1.495 (0.645 - 3.466)	0.349		
AFP, ng/mL (<400 *vs*. ≥400)	2.521 (1.442 - 4.408)	**0.001**	2.466 (1.405 - 4.326)	**0.002**	2.677 (1.196 - 5.989)	**0.017**	2.759 (1.216 - 6.261)	**0.015**
MVI (No *vs*. Yes)	2.076 (1.169 - 3.687)	**0.013**	1.825 (1.022 - 3.259)	**0.042**	0.663 (0.299 - 1.472)	0.313		
ALB, g/L (<35 *vs*. ≥35)	0.819 (0.435 - 1.542)	0.536			0.477 (0.210 - 1.081)	0.076		
Treatment (HAIC-L *vs*. HAIC-L-P)	0.509 (0.287 - 0.904)	**0.021**	0.530 (0.297 - 0.946)	**0.032**	0.618 (0.278 - 1.377)	0.239		

HCC, hepatocellular carcinoma; PSM, propensity score matching; DFS, disease-free survival; OS, overall survival; HR, hazard ratio; ALT, alanine transaminase; AFP, alpha-fetoprotein; MVI, microvascular invasion; ALB, albumin; HAIC, hepatic arterial infusion chemotherapy.

Bold values indicate statistically significant P values.

### 3.4 Safety

To compare safety profiles between groups post-PSM, treatment-related adverse events (TRAEs) were presented in **Table 3**. The overall incidence of TRAEs was similar between the HAIC-L and HAIC-L-P groups (any grade: 90% *vs*. 92%, P > 0.999; grade 1/2: 90% *vs*. 90%, P > 0.999; grade 3/4: 14% *vs*. 18%, P = 0.585). Most TRAEs were mild to moderate (grades 1-2), and no significant differences were observed in individual adverse events (P > 0.05). Notably, no treatment-related deaths or adverse events above grade 4 occurred in either group up to 12 months post-treatment. All adverse events resolved following symptomatic management or treatment discontinuation.

**Table 3:** Treatment-related adverse events of the patients with solitary large HCC after PSM.

Events, n (%)	HAIC-L (n=50)			HAIC-L-P (n=50)			P value		
	Any Grade	Grade 1/2	Grade 3/4	Any Grade	Grade 1/2	Grade 3/4	Any Grade	Grade 1/2	Grade 3/4
Any TRAE	45	45	7	46	45	9	>0.999	>0.999	0.585
Hematologic toxic effects									
Leukopenia	9	7	2	11	8	3	0.617	0.779	>0.999
Thrombocytopenia	4	4	0	7	7	0	0.338	0.338	>0.999
Hepatic function abnormalities									
Increased ALT	8	6	2	7	5	2	0.779	0.749	>0.999
Increased AST	6	5	1	6	4	2	>0.999	>0.999	>0.999
Hyperbilirubinemia	5	5	0	9	9	0	0.249	0.249	>0.999
Nonhematologic toxic effects									
Nausea	9	8	1	12	10	2	0.461	0.603	>0.999
Fatigue	11	9	2	13	11	2	0.640	0.617	>0.999
Fever	5	5	0	9	9	0	0.249	0.249	>0.999
Hypertension	7	7	0	4	4	0	0.338	0.338	>0.999
Pain	18	18	0	22	21	1	0.414	0.539	>0.999
Diarrhea	6	6	0	5	5	0	0.749	0.749	>0.999
Hypothyroidism	1	1	0	4	4	0	0.359	0.359	>0.999
Gastrointestinal hemorrhage	4	4	0	2	2	0	0.674	0.674	>0.999
RCCEP	0	0	0	3	3	0	0.241	0.241	>0.999

HCC, hepatocellular carcinoma; PSM, propensity score matching; HAIC, hepatic arterial infusion chemotherapy; ALT, alanine transaminase; AST, aspartate aminotransferase; RCCEP, reactive cutaneous capillary endothelial proliferation.

## 4 Discussion

To date, no universally accepted postoperative adjuvant therapy exists for HCC patients with high-risk features, and optimal strategies for solitary large HCC (>5 cm) remain underexplored. In this multicenter retrospective study, we demonstrated that postoperative adjuvant HAIC combined with lenvatinib and PD-1 inhibitors (HAIC-L-P) significantly improved DFS compared to HAIC plus lenvatinib alone (HAIC-L) in patients with solitary large HCC (>5 cm), both before and after PSM (HR: 0.570; P = 0.007; **Figure 2A**; HR: 0.518; P = 0.018; **Figure 3A**). Notably, the addition of PD-1 inhibitors led to a 48.2% reduction in progression risk after PSM, underscoring the potential of PD-1 inhibitors to augment the antitumor efficacy of combined locoregional and targeted therapy in this high-risk population. However, no significant OS benefit was observed between the two groups (P < 0.05). Multivariate analysis identified the treatment strategy (HAIC-L *vs*. HAIC-L-P; HR: 0.530; P = 0.032) as an independent predictor of DFS, reinforcing the clinical relevance of multimodal therapy in this setting.

The observed DFS benefit may arise from synergistic mechanisms between HAIC, lenvatinib, and PD-1 inhibitors. HAIC delivers high-dose chemotherapy directly to the liver, eliminating residual micrometastases and circulating tumor cells (11, 13). Recent studies have demonstrated the therapeutic efficacy and potential mechanisms of combining HAIC with PD-1 inhibitors (20, 21). Lenvatinib, a potent antiangiogenic agent, has been shown to promote vascular normalization and immune cell infiltration, thereby enhancing the efficacy of PD-1 inhibitors (22, 23). Furthermore, PD-1 inhibitors could reverse T-cell exhaustion and enhance immune surveillance against residual neoplastic clones. When combined with HAIC or anti-angiogenic therapy, these effects are further amplified, resulting in improved immune cell recruitment and function within the tumor milieu (20, 24, 25). This tri-modality approach aligns with emerging evidence in unresectable HCC, where HAIC/TACE combined with lenvatinib and PD-1 inhibitors has demonstrated superior tumor control (14, 26). Our findings extend these observations to the postoperative adjuvant setting, suggesting that combining locoregional chemotherapy, targeted therapy, and immunotherapy may disrupt the “seed-and-soil” interplay driving early recurrence in solitary large HCC.

A key observation in our study is the absence of an OS benefit despite the significant DFS advantage. This discrepancy, when contrasted with studies in advanced HCC where HAIC has frequently translated into prolonged OS (14, 27), may reflect inherent differences in tumor biology and treatment objectives. In the adjuvant setting, the aim is to eliminate micrometastatic disease. However, long-term survival after resection is also influenced by the availability and efficacy of salvage therapies following recurrence. Moreover, variations in patient characteristics and the underlying molecular and immunological heterogeneity of early-stage versus advanced HCC may contribute to these divergent outcomes. Recent comprehensive genomic analyses have identified distinct molecular subtypes of HCC with varying prognoses and therapeutic sensitivities (28). In our study cohort, it is plausible that PD-1 inhibitors effectively delayed recurrence in tumors with immunologically active microenvironments, thereby improving DFS. However, subsequent recurrences may have involved resistant clones or occurred in tumors with immunosuppressive features, leading to limited impact on OS. This hypothesis is supported by single-cell RNA sequencing studies revealing the dynamic evolution of immune cell states in HCC, including transitions toward exhausted or immunosuppressive phenotypes (29). Together, these factors highlight the complexities associated with translating DFS gains into OS benefits in the context of postoperative adjuvant therapy.

Multivariate analysis identified AFP ≥400 ng/mL and MVI as independent predictors of poor DFS, consistent with their established roles as biomarkers of aggressive biology and intrahepatic dissemination (30–32). The HAIC-L-P regimen appeared to mitigate the adverse prognostic impact of these factors, paralleling findings by Deng et al. (12), who reported that HAIC-based therapy reduced AFP levels more effectively than TACE in large HCC. The immunomodulatory effects of PD-1 inhibitors may further suppress AFP-secreting tumor subclones, warranting further investigation.

In our findings, safety profiles were comparable between the two groups, with no significant differences in all grade of TRAEs (P > 0.05). These findings demonstrated that both treatment approaches were generally well-tolerated, which consistent with previous studies (10, 33). The most common TRAEs were leukopenia, nausea, fatigue, and pain, which were consistent with known toxicities of HAIC, lenvatinib, and PD-1 inhibitors (15, 34). Notably, the addition of PD-1 inhibitors did not exacerbate hepatic toxicity, which is of particular concern in postoperative patients with compromised liver function.

Despite these promising findings, several limitations warrant consideration. First, the retrospective design of our study introduces inherent selection bias and unmeasured factors in treatment allocation, despite the use of PSM to minimize confounding. Second, the lack of biomarker data limits our mechanistic understanding of the immunotherapeutic response. Lastly, the relatively short follow-up period and small sample size may restrict the interpretation of OS outcomes. Future large-scale, randomized controlled trials are essential to validate these preliminary observations and to refine adjuvant therapeutic strategies for patients with solitary large HCC.

## 5 Conclusion

In conclusion, PA-HAIC combined with lenvatinib and PD-1 inhibitors represents a promising strategy for improving the DFS benefits in solitary large HCC, with a favorable safety profile. Future prospective trials with biomarker-driven designs and extended follow-up are warranted to validate these findings and optimize patient selection.

## Data Availability

The original contributions presented in the study are included in the article/supplementary material. Further inquiries can be directed to the corresponding authors.
